# Measurement of the Memory B Cell Response via Antibodies from Activated Cells

**DOI:** 10.3390/antib13040081

**Published:** 2024-10-03

**Authors:** Caroline Rockstroh, Katja Hintz, Judith Kannenberg, Christian Jassoy

**Affiliations:** Institute for Medical Microbiology and Virology, University Hospital and Medical Faculty, University of Leipzig, 04103 Leipzig, Germany

**Keywords:** memory B-lymphocyte, MBC enzyme-linked immunospot assay (ELISpot), enzyme-linked immunosorbent assay (ELISA), antibodies

## Abstract

Background/Objectives: The body’s immune response to infections and vaccination leads to the formation of memory B cells (MBCs), which protect against future infections. MBCs circulate in the blood, and the strength of the MBC response is measured with different tests. In this study, tests to measure the MBC response were compared. Methods: An MBC enzyme-linked immunospot assay (MBC-ELISpot), which counts the antibody-releasing cells (MASCs) that arise from memory B cells in vitro, and two versions of an MBC enzyme-linked immunosorbent assay (MBC-ELISA), which measures the concentration of antibodies released by the MASCs, were compared. The lower measurement limit of MBC-ELISpot and ELISA was determined, and it was investigated how the measurement results of individual samples and in a sample of test persons correlate. Results: Both methods had similar lower limits of detection, and the antibody concentration correlated strongly with the number of MASCs in individual samples. The antibody concentrations measured on a sample correlated with Pearson correlation coefficients of R = 0.83–0.87 with the number of MASCs, and the proportion of antigen-specific antibodies in total IgG correlated with R = 0.74–0.82 with the proportion of antigen-specific MASCs in all antibody-secreting cells. Conclusions: Since the measurement sensitivity of MBC-ELISA and MBC-ELISpot is similar and the measurement results correlate strongly in a random sample, the tests are interchangeable. The MBC-ELISA has an advantage over the ELISpot in that the antibody measurements can be divided up over time, repeated, and extended. This creates new possibilities for measuring the MBC response.

## 1. Introduction

The body reacts to infections caused by numerous pathogens with a specific immune response from T and B lymphocytes. This includes the formation of memory B cells (MBCs), which are designed to protect the body from future infections with pathogens. Some of these cells circulate in the blood. With suitable tests, antigen-specific memory B cells can be detected in the blood, the strength of the immune response can be quantified and MBC responses can be compared at different times and in different samples [1]. The challenge for such a test is to filter out the desired MBCs from the multitude of other MBCs in the blood and to set them in relation to all MBCs in the test sample. There are several methods for measuring the memory B cell response. The methods are briefly described here.

The cells in which the MBCs are found are first enriched by the density gradient centrifugation of the blood sample. The antigen-specific MBCs are then identified. In the direct MBC measurement, the cells are detected via the B cell receptor (BCR) that the MBCs carry on the cell surface. The BCRs correspond to the antibodies that the cell would synthesize and release after differentiation into a plasma cell. Fluorescent antigen molecules are added to the cell preparation, marking the MBCs to which the molecules bind. The proportion of antigen-specific MBCs can be calculated by simultaneously measuring the number of all MBCs [2,3,4].

In indirect MBC tests, the memory cells themselves are not measured. Instead, the MBCs are activated in vitro to differentiate into antibody-producing cells (ASCs), and the MBC response is detected and measured. There are two methods for quantifying antibody-producing cells that have developed from memory B cells (memory B-cell-derived antibody-secreting cells, MASCs): the limiting dilution method and the MBC-ELISpot. With the limiting dilution method, activated peripheral blood mononuclear cells (PBMCs) are seeded in varying numbers of cells in a 96-well culture plate. The culture plate is then examined to determine whether antibodies have been produced in the wells, and the proportion of MASCs among the total cells is calculated from the number of wells containing antibodies and the number of cells in them. In the ELISpot, PBMCs are stimulated for a few days, and the cells are then applied to an antigen-coated membrane in a 96-well culture plate. Antibodies can diffuse on the membrane and remain bound to the plate by the antigen, forming a halo around the antibody-secreting cells. The antibodies are visualized by immune-enzymatic treatment. Each spot represents one MASC. The spots are counted and compared with the number of cells used or the concentration of all antibody-secreting cells. The measurement results of the limiting dilution method and the ELISpot method correlate with each other [5].

In several recent articles, the concentration of antibodies in cell culture supernatants of activated PBMCs was used as a measure of the MBC response [6,7]. Nguyen-Contant and colleagues have reported that the antibody concentration in MASC cultures correlates with the number of MASC spots and can therefore be used as a measure of the size of the MBC population [6]. In the study by Long et al., this was shown in a small sample [8]. However, it has not yet been investigated whether the proportion of antigen-specific antibodies correlates with the proportion of MASCs in all antibody-secreting cells. As there are few data available in the literature on the comparison of antibody concentration and MASC numbers and the tests have not yet been examined with regard to the proportion of antigen-specific MBC response, the tests were compared again in this study.

## 2. Materials and Methods

### 2.1. Blood Samples, PBMC Preparation and MBC Activation

Blood from several healthy test subjects from studies about the SARS-CoV-2-specific memory B-cell response was used for the measurements [1]. A sample size calculation showed that 25 samples were required to determine whether the Pearson correlation coefficient differs from zero with an R-value of at least 0.6, a significance level of 0.05 and a power of 0.9 (https://sample-size.net/correlation-sample-size/ accessed on 30 September 2024). After informed consent, heparinized blood was collected, and PBMCs were isolated by Ficoll density gradient centrifugation. The cells were washed with PBS and resuspended in medium (RPMI-1640, 20% FCS, 100 U/mL penicillin, 100 µg/mL streptomycin, 1 mM sodium pyruvate, non-essential amino acids). The medium was supplemented with 1 µg/mL R848 (resiquimod, Sigma-Aldrich, Merck KGaA (Darmstadt, Germany) KGaA) and 0.11 µg/mL interleukin-2 (Proleukin, Novartis AG (Basel, Switzerland)). The cells were cultured for 5 days in a 24-well plate (Sarstedt AG & Co. KG, Nümbrecht, Germany) at 37 °C and 5% CO_2_. As a control, a portion of the PBMCs was cultured without R848.

### 2.2. Memory B Cell ELISpot

After 5 days of culture, the cells were transferred to prepared 96-well Multiscreen IP filter plates (Millipore, Merck KGaA, Darmstadt, Germany) and cultured again. The plates were first washed with 35% ethanol and PBS and then coated with 100 µL antigen or anti-IgG. The antigens used were selected for a study on the course of the SARS-CoV-2 memory B cell response and as controls [1]: tetanus toxin (Ttx, 5 µg/well, lot 317490, GSK Vaccines, Brentford, UK), influenza virus nucleoprotein as an MBP fusion protein (InfluNP, 2 µg/well) and recombinant SARS-CoV-2 RBD (RBD, 1 µg/well). The total number of IgG-secreting cells was determined using wells coated with mouse anti-human IgG mAb (clone MT91/145, Mabtech AB (Nacka Strand, Sweden), 5 µg/mL). Control wells were coated with MBP (1 µg/mL) or were uncoated. Plates were incubated overnight at 4 °C or for 2 h at 37 °C. Wells were washed with PBS and blocked for one hour with RPMI-1640 medium containing 20% FCS (R-20).

For antigen-specific MBCs, 300,000 stimulated cells in 200 µL were added to the antigen-coated wells and the control wells. To determine the IgG-secreting cells, 5000 cells were added to the anti-IgG-coated wells. The plates were incubated at 37 °C for 20 h. After incubation, the cells were removed, the plates were washed with PBS and PBS-T (PBS/0.05% Tween-20) and 100 µL/well of alkaline phosphatase (AP)-conjugated goat anti-human IgG (code no. 109-055-098, Jackson Immunoresearch Laboratories, Inc. (Cambridgeshire, UK), diluted 1:5000 in PBS) was added. The plate was incubated for 2 h at 37 °C, washed again and 100 µL/well NBT/BCIP substrate (AP Conjugate Substrate Kit, Bio-Rad Laboratories, Inc., Hercules, CA, USA) was added for 5 min. The plates were washed with water, dried overnight and read with the AID EliSpot/FluoroSpot Reader (AID Autoimmundiagnostika GmbH, Straßberg, Germany). Uncoated wells were used as negative controls for RBD and Ttx. Wells coated with MBP were used as negative controls for influenza NP.

### 2.3. Cell Culture Supernatants for Memory B Cell ELISAs

Two variants of the B cell memory ELISA were used. The first procedure was carried out in parallel with the ELISpot. For this purpose, 1250–10,000 (for total IgG-secreting MASCs) or 300,000 stimulated cells (for antigen-specific MASCs) were cultivated in 96-well cell culture plates in 200 µL/well R-20 at 37 °C for 20 h, and the cell culture supernatant was then obtained. In the second simplified procedure, cell culture supernatant was taken on day 5 from the cultures, with which the ELISpot was subsequently performed. Cell culture supernatant was also obtained from non-activated cells.

### 2.4. Antibody ELISAs

For the antibody ELISAs, 96-well microtiter plates (Greiner BioOne GmbH, Frickenhausen, Germany) were coated with goat anti-human IgA-IgG-IgM (Jackson Immunoresearch Laboratories, Inc., Art. No. 109-005-064, 1 µg/mL) or the Ttx (2 µg/mL), InfluNP (2 µg/mL) or RBD (1 µg/mL), diluted in PBS, pH 7.4, and incubated overnight at 4 °C. The plates were washed with PBS-T and treated with blocking solution (PBS-T, 5% milk powder) for 20 min. The blocking solution was removed, and concentration standards and cell culture supernatants diluted in blocking solution were added. The following reagents were used as concentration standards: polyclonal human IgG (Intratect, Biotest Pharma GmbH, Dreieich, Germany), 2nd International Standard Anti-Tetanus Immunoglobulin (WHO) NIBSC code 13/240 (https://nibsc.org/documents/ifu/13-240.pdf, accessed on 30 September 2024), monoclonal human anti-influenza virus nucleoprotein antibody SR2-NP66/67 [9], and human anti-SARS-CoV-2 RBD mAb CR3022 [10]. The plates were incubated at room temperature for 1 h. The liquid was discarded, and the plates were washed. Goat anti-human IgG antibody (Jackson Immunoresearch Laboratories, Inc., Art. No. 109-036-098, diluted 1:2000 for antigen-specific antibodies and 1:2000 or 1:5000 for total IgG) was added, and the plates were incubated for one hour at room temperature. The antibody solution was removed, the plates were washed again and tetramethyl benzidine (TMB, SeramunBlau slow 2 85, Seramun Diagnostica GmbH, Heidesee, Germany) was added for a few minutes. The color reaction was stopped by adding 0.5 M H_2_SO_4_. The light absorbance at 450 nm was measured with a photometer (Tecan Sunrise, Tecan Group Ltd., Männedorf, Switzerland).

### 2.5. Auxiliary Antigen-Specific Antibody Quotient and Statistical Analyses

We calculated a makeshift quotient from the specific antibody concentration and the total IgG, in which the units in which the antibody concentrations were measured were not taken into account. An auxiliary percentage was formed.

Curve fit analysis and calculation of the coefficient of determinations were performed with a built-in tool in the Microsoft Excel 2016 software. The Pearson coefficient of correlation was determined with the internet computer program Social Science Statistics (https://www.socscistatistics.com/tests/pearson/default.aspx, accessed 30 September 2024).

## 3. Results

### 3.1. Lower Limit of Quantification of the Antibody ELISAs

In order to produce antibody ELISAs with high analytical sensitivity, the concentration of the secondary antibody was maximized. At a dilution of the secondary antibody of 1:2000, the lower limits of quantification of the assays were up to IgG: 40 pg/mL, Ttx-IgG: 4 µU/mL, InfluNP IgG based on the SR2-NP66/67 antibody standard: 40 pg/mL, and SARS-CoV-2 RBD: 6 mBAU/mL.

### 3.2. Comparison of the Lower Detection Limits

**Figure 1:**
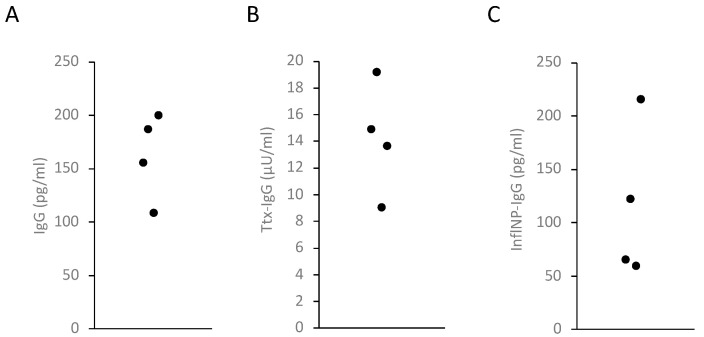
**Average antibody concentration per spot in a 200 µL cell culture.** Activated PBMCs from four individuals were cultured in a 96-well microplate and an ELISpot plate. Cell culture supernatants were harvested and the number of spots was counted. The supernatants were diluted according to the spot numbers and the antibody concentration was measured by ELISA. (**A**) IgG; (**B**) anti-tetanus toxin IgG antibodies; (**C**) anti-influenza virus NP IgG.

The MBC-ELISpot can be used to identify individual MASCs. To investigate whether the amount of antibodies secreted by a single MASC can be measured with the MASC ELISA, PBMCs from four individuals were stimulated and cultured in parallel in a 96-well ELISpot plate and in a cell culture plate. After 20 h, the incubation was stopped and the spots on the ELISpot plate were visualized and counted. The cell culture supernatants were removed from the cell culture plate, diluted according to the number of spots and measured in ELISAs. The antibody concentrations were in the measurement range of the ELISAs. The IgG concentrations per MASC varied between 100 and 200 pg/mL, the anti-Ttx antibodies were in the range of 9 to 19 µU/mL and the anti-InfluNP IgG concentration was between 65 and 216 pg/mL (Figure 1).

### 3.3. Correlation of Cell Count, Spot Count and Antibody Concentrations

**Figure 2:**
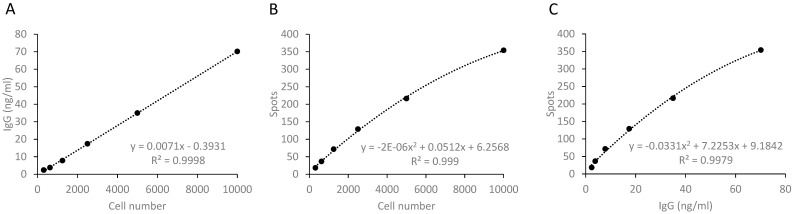
**Correlation between cell count, number of spots and IgG concentration.** (**A**) Regression line for IgG concentration and cell count; (**B**) regression curve for spot and cell count; (**C**) regression of spot count and IgG concentration.

To investigate the relationship between the antibody concentration and the number of MASCs in a broader measurement range, serial dilutions of 10,000 activated PBMCs were cultivated in cell culture and ELISpot plates for 20 h in parallel. The antibody concentration in the supernatants was measured and the MASC spots were counted. The antibody concentration correlated well with the PBMC number and the number of spots. Curve fitting of the values showed that the antibody concentration was linearly related to the cells used in the assay. The relationship between the spot count and the cell number and between the spot count and the antibody concentration was best represented by poly-nomial equations (Figure 2).

### 3.4. Correlation of Ttx-, InfluNP- and RBD-Specific Antibody Concentrations and Spot Counts

**Figure 3:**
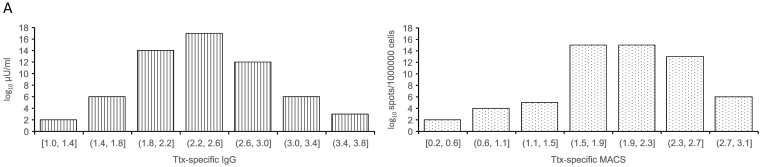

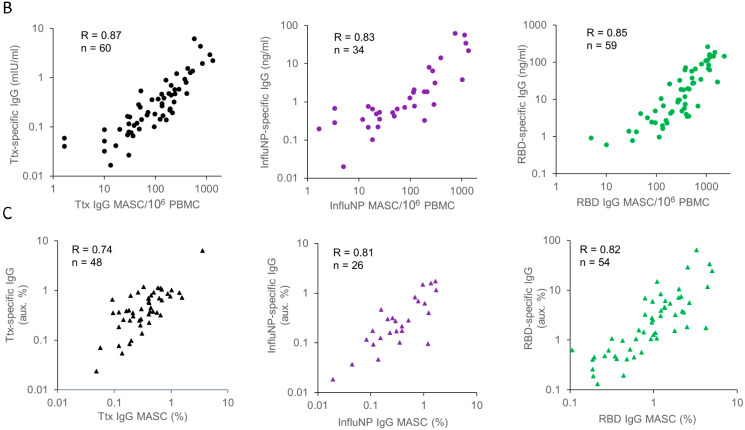
**Distribution and correlation of spot counts and antibody concentrations**. (**A**) Distribution of anti-tetanus toxin IgG concentrations and MASCs in 60 samples; (**B**) correlation of antibody concentrations and MASCs per 10^6^ PBMC; (**C**) correlation of the auxiliary proportion of the antigen-specific antibodies and the percentage of antigen-specific MASCs in all IgG-secreting cells. Aux. %: auxiliary antibody percentage, the value obtained by dividing the antigen-specific and total antibody concentrations by neglecting the different measurement units. R: Pearson correlation coefficient of the logarithmized values, *n*: number of samples.

The next step was to investigate the degree of accordance with a sample of test subjects. For this purpose, the Ttx-, InfluNP- and SARS-CoV-2-RBD-specific MBC response was measured on up to 60 blood samples from healthy individuals. We had previously found that 5 days after the activation of the PBMCs, the cell culture supernatants contained secreted antibodies. To simplify the MBC-ELISAs, the cell culture supernatant was harvested on day 5 and tested by ELISA. For the ELISpot, the cells were counted, cultured for a further 20 h and then the number of spots was determined. The antibody concentration and the number of spots in the samples were lognormally distributed. The antibody concentrations correlated strongly with the MASC numbers. The correlation coefficient R for the logarithmized antibody concentrations and MASC numbers per 10^6^ PBMC was 0.83–0.87. When comparing the proportion of antigen-specific IgG using auxiliary percentage values with the percentage of antigen-specific MASCs in all IgG-releasing MASCs, the correlation analysis yielded R values of 0.74–0.82 (Figure 3).

## 4. Discussion

Among other parameters, the B cell memory cell response is considered a measure of B cell immunity after infections and vaccinations. There are several methods for measuring the MBC response. One commonly used method is the MBC-ELISpot, in which MASCs are counted and the MBC response is inferred [5]. Another method is the MBC-ELISA, in which the antibody concentration in cell culture supernatants of MASCs is measured and used as a measure of the MBC response [6,8,11]. In this study, the two methods were compared with each other. The amount of antibody secreted by a single MASC during 20 h was detectable by the MASC ELISA. The spot count and the antibody concentrations correlated well when the tests were performed in the same way. The observations indicate that, in principle, the two tests have the same lower level of quantification and make similar statements about the MBC response.

For practical application, the MBC-ELISA was simplified by obtaining the cell culture supernatants of the activated PBMC without re-counting and re-culturing the cells. This test was tested with three antigens together with the MBC-ELISpot on a larger number of blood samples. The antibody concentrations correlated strongly with the MASC numbers. These results are consistent with the observations of Long et al., who investigated the MBC response against SARS-CoV-2 RBD in SARS-CoV-2-infected individuals [8]. Together, the data indicate that the two tests are to some extent interchangeable.

There are various, slightly different methods for the activation and culture of MBCs in the literature, but they are presumably equally suitable for the MBC-ELISA [6,7,8,11]. Thorough washing of the PBMCs is necessary to completely remove the antibodies present in the serum. The study was conducted with memory B cells for three antigens that were easy to analyze. However, we believe that the observations also apply to the measurement of the memory B cell response to other antigens.

When comparing the spot number with other values, Figure 2 shows a deviation of the regression curve from a straight line to a curve that takes the form of an asymptote. This is an expression of the fact that the accuracy of the determination of the spot number decreases with high spot numbers because spots merge with each other. This makes the exact determination of the spot number imprecise with high spot numbers. In comparison, the antibody concentration can be accurately determined over a wider concentration range.

When measuring the MBC response in a sample, the MASCs are often not only counted but the proportion of antigen-specific MASCs in all antibody-secreting cells is calculated [5]. This takes into account that the proportion of MBCs differs slightly between individuals and increases with age [12]. For this purpose, antigen-specific spots and all IgG-secreting cells in a sample are measured and the quotient is calculated. In order to adapt this to the antibody concentrations, a makeshift quotient was formed. The quotient does not take into account that the units in which the antigen-specific and total antibodies were measured are different. For example, the quotient was formed from tetanus antibodies and total IgG, ignoring the units µU and ng. It should be noted that the ratios do not reflect the actual proportion of antigen-specific antibodies due to the different units. Auxiliary percentages were formed from the quotients. The percentage of antigen-specific antibodies in the total IgG correlated strongly with the proportion of antigen-specific MASCs in all MASCs. We conclude that the two tests are interchangeable when studying the MBC response in groups of subjects.

Compared to the MBC-ELISA, the MBC-ELISpot has the disadvantage that it can only be performed once on a sample and in a short time window after PBMC activation. As the MBC-ELISA is carried out with cell culture supernatant, it is independent of the cell cultures in terms of time and place. Measurements can be repeated if necessary and further MBC tests can be carried out subsequently. For example, the MBC response against other antigens or new antigen variants can be subsequently investigated.

## 5. Conclusions

In summary, MBC-ELISA and ELISpot measure the MBC response in different ways but come to the same result. The two methods therefore complement each other or are interchangeable. The MBC-ELISA offers the advantage that the measurements can be repeated and the range of tests can be extended retrospectively.

## Data Availability

Original data on the experiments can be made available upon request.
